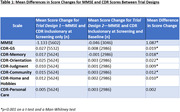# MMSE and CDR Score Changes and Potential Score Inflation in Multinational Alzheimer’s Disease Trials

**DOI:** 10.1002/alz.089777

**Published:** 2025-01-09

**Authors:** Amanda Hackebeil, Gila Barbati, Sayaka Machizawa, Jessica Stenclik, Erica R. Appleman, Andrei Iacob, Jacqueline Massa

**Affiliations:** ^1^ Signant Health, Blue Bell, PA USA

## Abstract

**Background:**

In Alzheimer’s Disease trials, the Mini‐Mental State Examination (MMSE) and Clinical Dementia Rating (CDR) are commonly utilized as inclusionary criteria at screening. These measures, however, do not always reaffirm inclusionary status at baseline. Score changes between screening and baseline visits may imply potential score inflation at screening leading to inappropriate participant enrollment. This study compared score changes in global AD trials when MMSE and CDR scores were used as inclusionary measures at screening only versus screening and baseline visits.

We hypothesized greater score changes would be observed in trials utilizing these inclusionary measures at screening alone.

**Method:**

This study incorporated electronic scale (eScale) data from three global Phase 3 trials comprised of MCI to Mild AD participants, where the MMSE and CDR were used as inclusionary criteria. Two studies applied inclusion scoring criteria at screening, while one study applied inclusion scoring criteria at both screening and baseline. All raters completed Signant Health standardized rater training and certification programs. eScales and data quality monitoring programs increased rater scoring accuracy. Mann‐Whitney U tests and t‐tests were conducted comparing score changes on MMSE total score and CDR Domain and Global Scores.

**Result:**

Both Mann‐Whitney U and t‐tests revealed significant differences in score changes between screening and baseline visits for the two trial designs, including all domain and total scale scores, except for the CDR Personal Care domain (p<0.001), as seen in Table 1. Increased scores for the CDR domain/Global scores and reduced scores on MMSE were observed when inclusionary criteria were required at screening alone.

**Conclusion:**

The present study found greater score changes in the CDR and MMSE for participants who met inclusion criteria at screening only compared to both screening and baseline. These findings suggest potential score inflation at inclusionary visits when inclusionary scoring is required only at screening. To reduce inclusionary bias and inappropriate participant enrollment, a data quality surveillance program is recommended at inclusionary and subsequent visits throughout the study. Future studies should explore additional factors contributing to score change.